# Nucleation of amorphous shear bands at nanotwins in boron suboxide

**DOI:** 10.1038/ncomms11001

**Published:** 2016-03-22

**Authors:** Qi An, K. Madhav Reddy, Jin Qian, Kevin J. Hemker, Ming-Wei Chen, William A. Goddard III

**Affiliations:** 1 Department of Chemistry, Materials and Process Simulation Center, California Institute of Technology, 1200 East California Boulevard, Pasadena, California 91125, USA; 2 WPI Advanced Institute for Materials Research, Tohoku University, Sendai 980-8577, Japan; 3 Department of Mechanical Engineering, Johns Hopkins University, Baltimore, Maryland 21218, USA

## Abstract

The roles of grain boundaries and twin boundaries in mechanical properties are well understood for metals and alloys. However, for covalent solids, their roles in deformation response to applied stress are not established. Here we characterize the nanotwins in boron suboxide (B_6_O) with twin boundaries along the 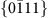 planes using both scanning transmission electron microscopy and quantum mechanics. Then, we use quantum mechanics to determine the deformation mechanism for perfect and twinned B_6_O crystals for both pure shear and biaxial shear deformations. Quantum mechanics suggests that amorphous bands nucleate preferentially at the twin boundaries in B_6_O because the twinned structure has a lower maximum shear strength by 7.5% compared with perfect structure. These results, which are supported by experimental observations of the coordinated existence of nanotwins and amorphous shear bands in B_6_O, provide a plausible atomistic explanation for the influence of nanotwins on the deformation behaviour of superhard ceramics.

## Abstract (web summary)

Grain boundaries affect the physical properties of metals but their influence on covalent solids is less well established. Here, the authors use scanning transmission electron microscopy and quantum mechanics to understand deformation mechanisms in perfect and twinned boron suboxide crystals.

## Graphical abstract

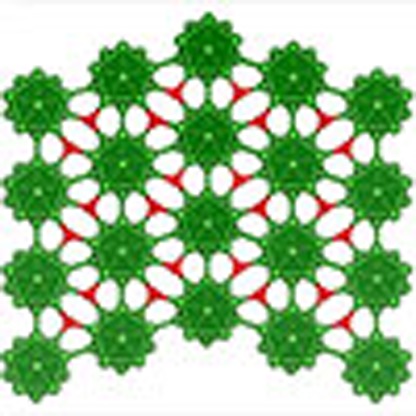

Grain boundaries (GBs) and twin boundaries (TBs) are known to strengthen polycrystalline materials such as metals and ceramics, where GBs can impede dislocation movements, so that decreasing grains sizes can strengthen the material, the Hall–Petch relationship^1^,^2^,^3^,^4^. However, for grain sizes below some critical value, the dominant deformation mechanism becomes GBs sliding and migration so that the material strength decreases for further decreases in grain size^5^,^6^. These grain size effects have been studied extensively in metals but not in bulk nanocrystalline ceramics^7^,^8^. Dislocation migration so important in metals is absent in most ceramics, especially at low temperature, so that we can expect quite different roles of GBs and TBs in the deformation mechanisms of ceramics as compared with metals and alloys.

Nanotwins are expected to be much lower in energy than normal GBs and might strengthen materials more efficiently^9^. For metals, nanotwinned systems generally fail well below their theoretical strength limit because of dislocation nucleation and emission from boundaries or surface imperfections^10^. However, in ceramics, nanoscale twins have been observed to dramatically enhance the hardness of diamond and boron nitride^11^,^12^, inspiring our examination of the roles of nanotwinned structures on the deformation mechanism in ceramics and covalent solids.

As a prototype model system for examining how twin structures affect the mechanical response under deformation, we examined superhard boron suboxide (B_6_O) ceramic because B_6_O is capable of forming unusual profusely twinned crystals with icosahedral habits^13^. In addition, B_6_O is a promising hard material with a Vickers hardness of 45 GPa, which can be used in manufacture of cutting tools, abrasives and body armour^14^,^15^ (also quantum mechanics (QM) calculations predict a direct band gap of ∼3.0 eV, making it a candidate for a *p*-type transparent conducting oxide^16^). B_6_O has been observed experimentally to undergo brittle failure with formation of shear-induced amorphous bands^17^ similar to boron carbide (B_4_C)^18^,^19^. We previously reported QM and reactive force field molecular dynamics simulations showing that brittle failure in B_4_C arises from formation of higher density amorphous bands that result from fracturing the icosahedra because of reactions with the distorted C–B–C chains^20^,^21^. The intrinsic failure mechanism of B_6_O is not known. However, in B_6_O, the chains connecting the icosahedra involve two weakly coupled O atoms so that we expect that the icosahedra might not fracture under pure shear conditions^22^.

In this study, we use the state-of-the-art spherical aberration (Cs) corrected scanning transmission electron microscopy (STEM) to examine the nanoscale twins found in hot pressed B_6_O, showing that the TBs lie on the 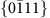 family planes and the angle between 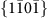 and 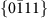 planes is 72.0°±0.3°. Then we use QM at the Perdew–Burke–Ernzerhof density functional theory (DFT) level to predict the most favourable twins. We find them to be in the 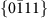 family plane with angles of 72.0°, in excellent agreement with our experiments. Next, we used QM to perform shear deformation along the 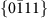 twin plane, where we find that the maximum shear strength for shearing twinned B_6_O is 43.3 GPa, very close to the ideal shear strength of 43.5 GPa for single crystal B_6_O, and higher than values for perfect B_4_C (39.0 GPa)^20^. Finally, we performed biaxial shear deformation to mimic indentation experiments for both perfect and twinned B_6_O. The DFT simulations suggest that indentation conditions lead to deconstruction of the icosahedra, providing a plausible explanation for the amorphous band formation observed experimentally in B_6_O. We predict that the maximum shear stress is 7.5% lower for twinned B_6_O, and that amorphous bands might nucleate at the nanoscale twins, which are supported by experimental observations of the coordinated existence of nanoscale twins and amorphous shear bands. These results indicate that TBs play an essential role in the deformation mechanism and intrinsic brittle failure of B_6_O.

## Results

### Perfect and nanotwinned B_6_O structures

**Figure 1:**
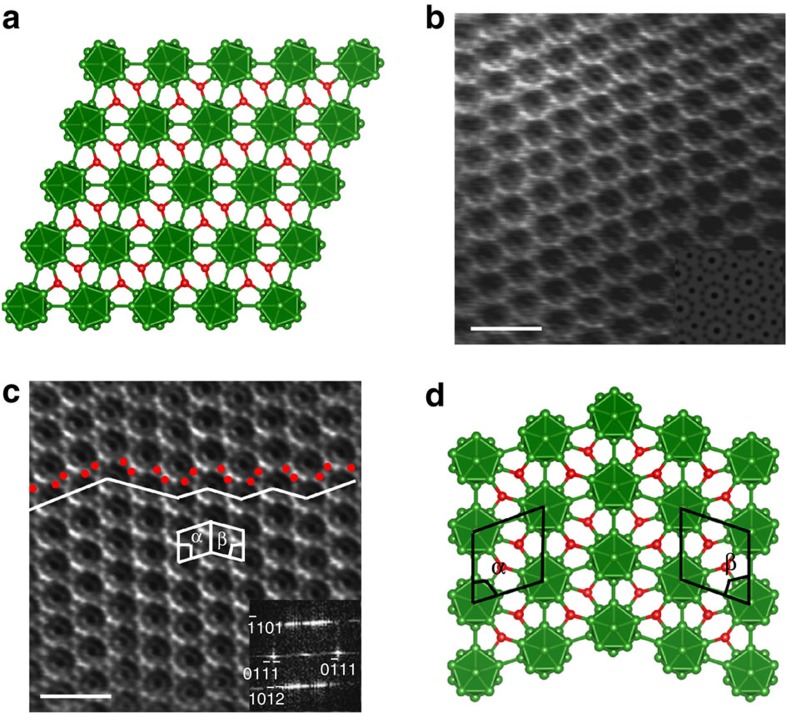
The perfect and twinned B_6_O structures from QM and STEM images. (**a**) The B_6_O rhombohedral structure, icosahedral B_12_ clusters (green colour) and linked oxygen atoms (red colour). (**b**) The annular bright-field STEM (ABF-STEM) image showing the B_12_ icosahedra as atomic rings linked between two oxygen atoms along 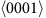 direction. The simulated ABF-STEM image of B_6_O is inserted at the bottom side. (**c**) A representative ABF-STEM image showing highly dense nanoscale twin bands within a grain and along the 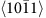 zone axis. Images have been smoothed and Fourier filtered to enhance the contrast of nano-twinned B_6_O atomic structure (see Supplementary Fig. 9). (**b**,**c**) Scale bars, 1 nm. (**d**) The twinned B_6_O structure from QM. The measured angles on both sides of the TBs are *α*=72.0° and *β*=72.0°, which are consistent agree well with the experimental values of 72.0°±0.3° as shown in FFT pattern (inset **c**).

The crystalline structure of B_6_O consists of one B_12_ icosahedron per rhombohedral unit cell (space group *R*
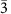
*m*), as shown in Fig. 1a, which is similar to B_4_C, B_12_P_2_ and *α*-boron^22^. The B_12_ icosahedra are connected via pseudo chains consisting of a pair of oxygen (O–O) atoms along the 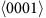 direction. Here each O makes B–O sigma bonds to three icosahedra, but the O–O distance of 3.07 Å is far too long for a bond. Thus, each O is formally O^+^, transferring one electron to a B_12_ icosahedron, leading to 26 skeleton electrons within the icosahedron to satisfy Wade's rule^23^. Each icosahedron also makes six direct B–B sigma bonds to adjacent icosahedra. Annular bright-field STEM (ABF-STEM; Fig. 1b) shows the atomic image of perfect structure of B_6_O along the 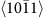 projection. Comparing the experimental and computer simulated ABF-STEM images (inset bottom right) reveals 12-atom boron icosahedra (B_12_) in the form of atomic rings with two oxygen atoms distinguishably appearing as a dark-dot contrast linking the icosahedra. This can be recognized as oxygen atomic columns positioned to be consistent with the structural model of B_6_O in Fig. 1a. Figure 1c shows representative nanoscale twins observed with ABF-STEM showing that the twin bands range in width from sub-nanometre to a few nanometres within the B_6_O grains (white lines). The TBs lie on the 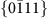 family planes, which agree very well with previous studies on twinned B_6_O and B_4_C (refs 13, 24, 25, 26). In addition, the ABF-STEM image and inset fast Fourier transform (FFT) pattern demonstrate clearly that the angles between 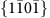
and 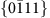 planes are 72° on both sides of the TBs, indicating symmetric twins in B_6_O.

To interpret the experimentally observed twinned structure in B_6_O, we used QM to determine the atomic geometry of the twin, leading to the results in Fig. 1d. Our QM unit cell considered total four layers of icosahedra perpendicular to the 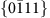 twin plane, leading to two TB planes per unit cell. We also constructed an eight-layer twinned structure to examine the multiple layer effects. These QM twin models can be compared with experimental structures having only several layers of icosahedra, as shown in Fig. 1c. The QM predicts angles on both sides of the TBs of *α*=72.0° and *β*=72.0° in excellent agreement with our experimental value of 72.0°±0.3°. Moreover, the QM predicts an interfacial energy for the TB of only −1.69 mJ m^−2^, which is much smaller than the stacking faults energy in most metals and ceramics (>20 mJ m^−2^)^27^,^28^. This low interfacial energy may explain the unusual highly twinned crystals that we found in the experimentally synthesized B_6_O.

### Deformation mechanism of nanotwinned B_6_O

**Figure 2:**
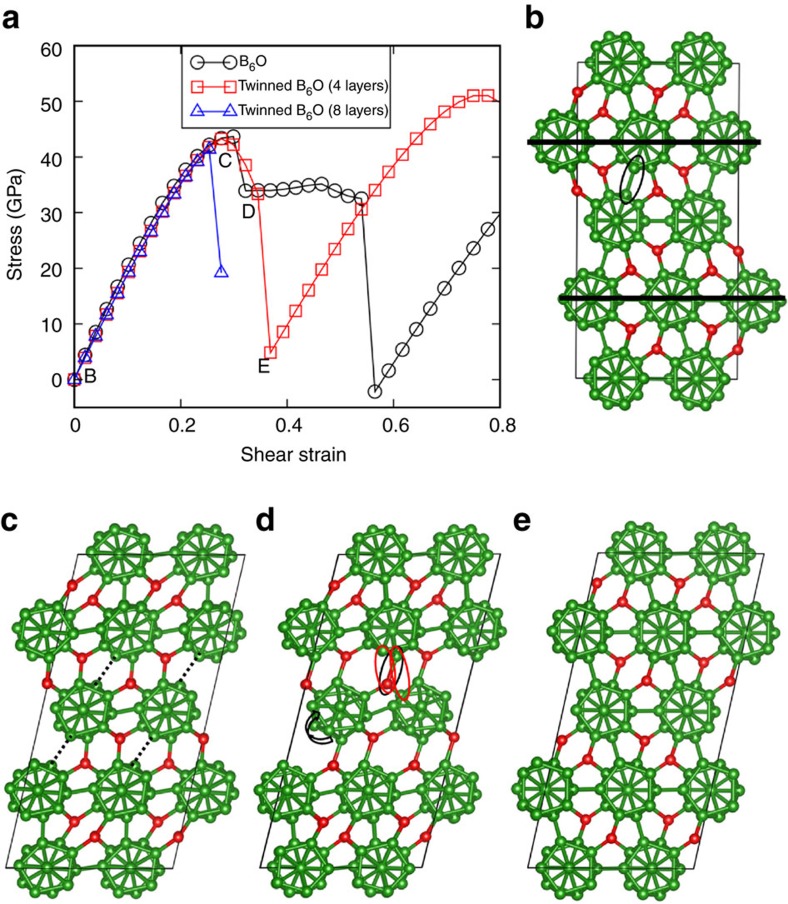
Pure shear deformation along the {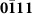} twin plane for the twinned structure compared with perfect B_6_O. (**a**) Stress–strain relationship. (**b**) Initial twin structure at 0.0 strain. The twin boundary is represented by solid black line. The B–B bond within the oval breaks in the following shear steps. (**c**) Structure at 0.276 strain, which corresponds to the maximum stress. The stretched B–B bonds are represented by dashed lines. (**d**) Structure at 0.345 strain, before the structure transformation. The donor–acceptor B–O bond is within the black oval and the forming B–O and B–B bond are within the red ovals. (**e**) Structure recovery to perfect crystal at 0.369 strain.

The existence of large numbers of nanoscale twins in B_6_O might be expected to dramatically change the mechanical properties, especially the strength and failure mechanism. To investigate these effects, we used QM to describe the effect of shearing the twinned supercell B_6_O parallel the 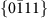 twin plane. The computed stress–strain relationship for the twinned structure is shown in Fig. 2a and is compared with that of the perfect B_6_O (ref. 22). The slope of the stress–strain curve for the twinned structure is 7.5% lower than that of perfect B_6_O, confirming the softening effect of the TBs. The maximum shear stress is 43.3 GPa for the twinned structure, very close to our calculated ideal shear stress of 43.5 GPa (ref. 22). This indicates that the nanotwinned structure could have the theoretical strength. It is well known that B_6_O is anisotropic and the lower strength for twinned structure might arise from the anisotropic effects. Thus, we also sheared the perfect crystal along the 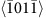 (opposite direction of 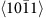) and obtained a higher maximum shear stress of 51.0 GPa because of the directional of covalent bonding in B_6_O. This indicates that the twinned structure has intrinsically lower strength than perfect B_6_O.

As the twinned structure is sheared, we observed that the middle layers (Fig. 2b) start to shear along the 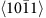 easy direction. However, the other layers shear along the opposite hard shear direction of 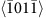. As the shear stress increases to its maximum at 0.276 strain, a B–B bond (black oval in Fig. 2b) between icosahedra increases from 1.72 Å in the intact structure (Fig. 2b) to 2.31 Å (Fig. 2c). To analyse the bonding, we use the electron localization function^29^ shown in Supplementary Fig. 1a,b, which indicates that the electron pair in the B–B covalent bond is not broken until the shear strain increases to 0.345 (Fig. 2d). After breaking the B–B bond, the icosahedra in the middle layer (indicted by the black arrowhead in Supplementary Fig. 1b) have only ten extra-cage bonds so that they do not need extra electrons from nearby O atoms to satisfy Wade's rule. The black oval in Fig. 2d shows that the B–O bond distance increases from 1.51 to 1.64 Å, indicating that some covalent B–O bonding changes to donor–acceptor character. In the starting structure, the O is effectively O^+^, which like N can make three covalent bonds, leaving a lone pair pointing at the other O; but for the strained system, the O is neutral so that it can make two covalent bonds and one donor–acceptor (Lewis base–Lewis acid) bond, which get averaged over the three B–O linkages. The icosahedra near the middle layer (indicated by the red arrowhead in Supplementary Fig. 1b) now also have ten extra-cage bonds because one B–O bond becomes a donor–acceptor bond with only one broken B–B bond between icosahedra. This leads to a slight rotation of the icosahedral cluster in the middle layer, as shown in Fig. 2d. Finally, as the shear strain increases continuously to 0.369, the donor–acceptor B–O bond breaks, a new B–B covalent bond forms between the cages, and a B–O covalent cage chain bond forms (red ovals in Fig. 2d). The result is to reconstruct the perfect crystal structure, as shown in Fig. 2e.

It is of interest to determine whether structure recovery happens at finite temperature for fast strain rates. To address this issue, we performed *ab initio* molecular dynamics (AIMD) simulations assuming constant volume shear deformation along the same slip system at room temperature using the strain rate of 1.0 × 10^10^ s^−1^. Here we fixed the volume in the MD simulations to mimic the high strain rate deformation. The stress–strain relationship and the structural changes displayed in Supplementary Fig. 2 show that there is structure recovery. Thus, we find that the original twinned structure recovers back to twinned structure, which is different from what we find for static shear deformations where the twinned structure transforms to a perfect crystal structure. Thus, we conclude that the twinned structure will recover to itself during high strain rate shear deformations at room temperature.

To examine how the multiple layers between TBs might affect the deformation mechanism described above, we constructed the eight-layer twinned structure as shown in Supplementary Fig. 3a and applied a pure shear deformation to it. The stress–strain relationship in Fig. 2a shows that the maximum shear stress decreases from 43.3 to 41.4 GPa as the number of layers between TBs increases from 2 to 4, indicating a weakening effect because of the multiple layer structure. The structural changes for this eight-layer twinned structure under pure shear deformation are displayed in Supplementary Fig. 3b,c. As the shear strain increases to 0.254, which corresponds to the maximum shear stress, the B–B bonds between icosahedra (within the region of the easy shear direction) break, as shown in Supplementary Fig. 3b. As the shear strain increases to 0.276, the middle two layers within the easy shear region recover to the crystal structure with the direction along the hard shear direction (the other twinned region). This leads to one-layer twin structure (or stacking fault) within a new eight-layer structure, as shown in Supplementary Fig. 3c. No icosahedra break in this shear process, which is similar to the four-layer twinned and perfect B_6_O structures^22^.

Although the twinned structure has intrinsically lower strength than perfect B_6_O, the eight-layer twinned structure is even weaker than four-layer twinned structure. In the sheared deformation of the twinned structure, the half layer is sheared along the 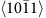 easy direction, whereas the other half layer is sheared along the hard shear direction of 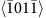. In the four-layer twinned structure, both the two easy sheared layers are directly bonded to the two hard sheared layers, which make the TBs harder to slip than eight-layer twinned structure where only the boundary layers are bonded to hard sheared layers.

Our simulations suggest a weakening effect from TBs compared with the ideal strength of the perfect crystal. The ideal strength of single crystals is relevant only for incipient plasticity. Previous experimental studies infer the hardening effects of twins in diamond and *c*-BN^11^,^12^ based on the hardness measurements under conditions of fully developed plasticity. No ideal crystal exists in these conditions. Further study is required to understand the twin hardening mechanism for diamond/*c*-BN under experimental conditions.

The shear simulations on both twinned B_6_O and perfect B_6_O lead to structural recovery without breaking the icosahedra. This suggests a critical design element for designing ductile hard materials of boron suboxide and related materials. The key is to form donor–acceptor bonds between the icosahedra and chain structures that can break the cage–chain bonds to relax the high stress without breaking the icosahedra. This prevents amorphous band formation, leading to improved ductility of superhard ceramics.

### Nano-indentation on nanotwinned B_6_O

To characterize the shear deformation of B_6_O experimentally, we carried out nano-indentation experiments on highly dense nanotwinned bulk specimens and prepared a cross-sectioned thin foil of the area beneath the indent using FIB milling. Conventional transmission electron microscopy (TEM) and STEM observations were used to examine the underlying mechanism.

**Figure 3:**
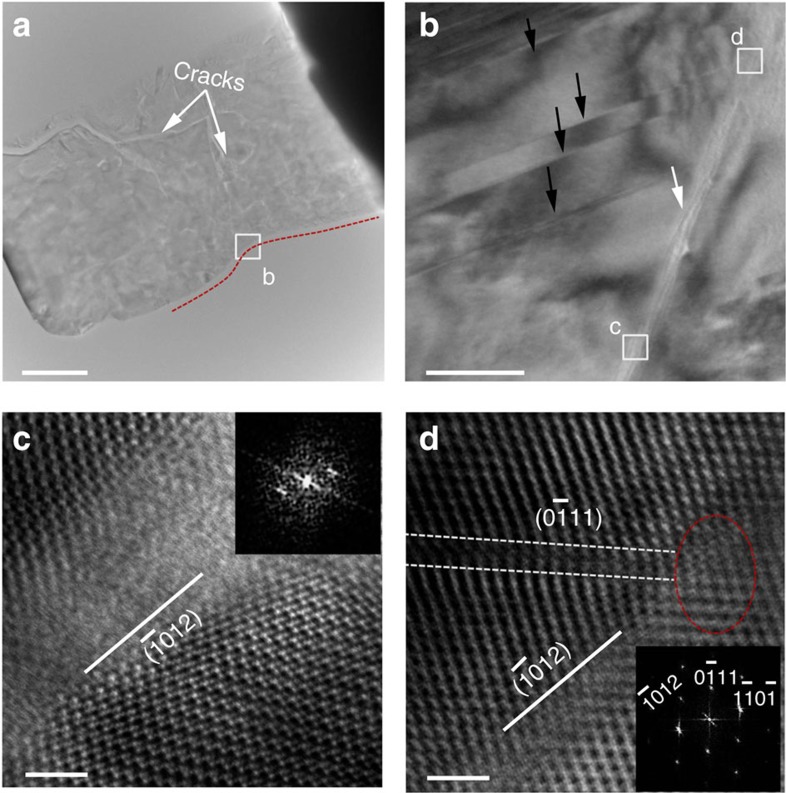
TEM images of the cross-sectioned indentation region. (**a**) Low-magnification TEM beneath the indentation region. Scale bar, 2 μm. (**b**) Magnified TEM image beneath the indented region showing a shear band (white arrowhead) in the highly dense twin grain (black arrowhead) from the 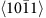 projection. Scale bar, 50 nm. (**c**) ABF-STEM taken from the shear band reveals the loss of periodicity in a B_6_O grain. Inset FFT pattern demonstrate the amorphous nature of the shear band. The crystalline of B_6_O can be found on both sides of amorphous band. (**d**) ABF-STEM image at the tip of amorphous shear band (red circle) shows dissociated icosahedral clusters initiated from a twin bands. The white box is to help distinguish twin structure. (**c**,**d**) Scale bar, 2 nm.

Figure 3 shows the TEM and STEM images of the indentation region of B_6_O. The low-magnification TEM image (Fig. 3a) shows several micro-crack regions and amorphous shear bands, suggesting that severe plastic deformation and damage take place underneath the indenter. The higher magnification TEM images (Fig. 3b and Supplementary Fig. 4) indicate that the nanoscale amorphous band was formed in close proximity to and intersects numerous nanoscale twins. The amorphous band is 200–300 nm long with a 1- to 3-nm width. The nanoscale twin bands and the amorphous shear band are highlighted with black and white arrowheads in Fig. 3b–d). The atomic structure of the amorphous B_6_O band, viewed along the 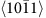
zone axis, is shown in Fig. 3c; crystalline B_12_ icosahedra are visible on both sides, but a loss of periodicity is evident within the ∼3 nm band. A high-resolution STEM image of this amorphous band is shown in Fig. 3d and indicates that amorphous B_6_O band formation occurs along the 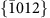 plane and intersects at their close proximity of observed nanoscale twin plane. In Fig. 3c,d, white dots correspond to the central positions of the B_12_ icosahedral clusters as suggested by image simulation^30^. In Fig. 3d, the amorphous band is less than 1 nm in width, as compared with 3 nm in Fig. 3c. The measured angle between the amorphous band plane and the twin plane is about ∼54°.

Our previous study^21^ showed that the amorphous band has higher density than the crystalline region because of deconstruction of the icosahedral clusters. This leads to the severe lattice distortion in the local amorphous region that likely causes the different angles between 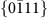 planes compared with the intact crystal. The FFT patterns in Fig. 3d from localized region around amorphous band have angular mismatch ∼3° of crystals on either side of the amorphous band as compared with predicted angle. However, selected area diffraction patterns taken from large areas (few hundred nanometres) on either side of amorphous band as shown in Supplementary Fig. 4 are consistent with the theoretical angle of 72.0° of 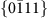 planes. These observations confirm the severe deformation of crystalline region near the amorphous band in B_6_O.

### Deformation mechanisms under indentation

**Figure 4:**
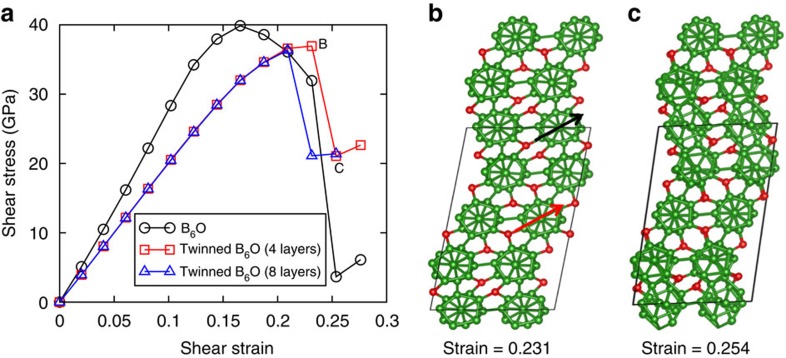
Biaxial shear deformation of twinned B_6_O shearing along the {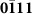} twin plane to mimic an indentation experiment, which is compared with the perfect B_6_O. (**a**) Stress–strain relationship. (**b**) Structure at 0.231 strain before failure. The long axis of the most distorted and the least icosahedra are represented by black and red arrowhead, respectively. (**c**) Structure at 0.254 strain after failure.

To understand the amorphous shear band formation mechanism observed in the indentation experiments, we used DFT to perform biaxial shear deformations. The stress–strain relationships under biaxial shear deformation for both twinned and perfect B_6_O are shown in Fig. 4a. In contrast to pure shear deformation, we calculate that the slope of the stress–strain relationship for twinned structure is 23.4% smaller than the perfect B_6_O, indicating that TBs under indentation induce a pronounced softening effect. The maximum shear stress for twinned B_6_O is 36.9 GPa, which is 8% lower than for the perfect B_6_O (39.9 GPa). As we discussed in the pure shear deformation, the twinned structure has intrinsically lower strength than perfect B_6_O because of interfacial energy from twins. This suggests that amorphous bands might nucleate at the TBs under indentation conditions. Other defects such as vacancies and GBs might also be important in the experiments on amorphous band formation.

Under biaxial shear conditions, we find that compression perpendicular to the TBs plays an essential role in the disintegration of icosahedra. As the shear stress reaches its maximum value of 36.9 GPa at 0.231 strain, no bonds break, as shown in Fig. 4b. Instead, the icosahedra deform to an ellipsoid shape that accommodates the applied biaxial stress. The most deformed icosahedra are in the layer that shears along the hard direction. This can be characterized by the B⋯B distance (opposite to each other in the cage, indicated by black arrowhead in Fig. 4b), which increases from 3.39 to 3.66 Å. In contrast, this distance is 3.60 Å for the least distorted icosahedra located in the layer (red arrowhead in Fig. 4b) that is shearing along the easy direction. This deconstructs the icosahedra layers along the hard shear directions as the strain increases to 0.254, as shown in Fig. 4c.

To examine how the multiple layers between TBs affect the biaxial deformation mechanism, we applied biaxial shear deformation to the eight-layer twinned structure. The maximum shear stress for the eight-layer twinned B_6_O is 36.3 GPa, which is very slightly lower than the four-layer twinned structure of 36.9 GPa, as shown in Fig. 4a. The biaxial deformation mechanism for eight-layer twinned structure is similar to four-layer twinned structure where the icosahedra within the hard shear direction are disintegrated under the effects of both compression and shear, as shown in Supplementary Fig. 5.

The structural changes of perfect B_6_O under indentation conditions are shown in Supplementary Fig. 6a–d. As the shear stress reaches its maximum of 39.9 GPa at 0.166 strain, the icosahedra deform without breaking any bonds (Supplementary Fig. 6a). The B–B bond between icosahedra along the 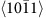 direction stretches from 1.70 to 1.99 Å. As the shear strain continuously increases farther to 0.231, the shear stress decreases to 31.9 GPa. The icosahedra deform to an ellipsoidal shape (Supplementary Fig. 6b), where the B⋯B distance for the long axis along the 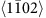 direction (opposite to each other along the 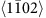) increases to 3.89 Å. This leads to deconstruction of icosahedra as the shear strain increases to 0.254, as shown in Supplementary Fig. 6c. Not all icosahedra in the simulation cell break. Instead, the icosahedra deconstruct along the alternative layers of 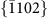 planes, as shown in the rotated structure of Supplementary Fig. 6d.

## Discussion

**Figure 5:**
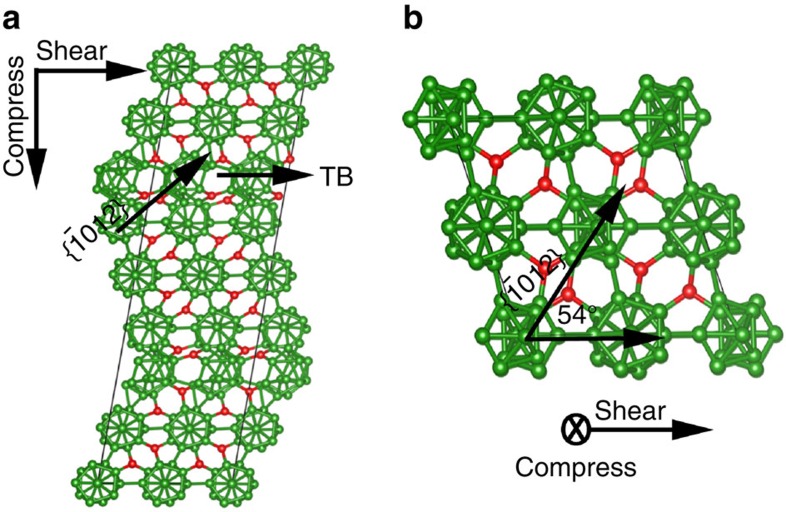
Deformation mechanism for forming an amorphous shear band at realistic experimental conditions. (**a**) The biaxial shear deformation of four-layer nanotwins leads to deconstruction of icosahedra along the TB plane, corresponding to the red circle in Fig. 3d. (**b**) The biaxial shear deformation of perfect crystal leads to deconstruction of icosahedra along 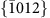 plane, which aligns the amorphous band propagation in Fig. 3c,d.

Combining the experimental TEM observations with the QM-derived deformation mechanism under biaxial deformation, we propose a deformation mechanism in which the amorphous band initiates at the nanoscale twins and then it propagates along the 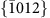 family plane, as shown in Figs 3 and 5. The process can be described as follows. First, two layers of icosahedra along the TBs disintegrate under the indentation conditions, as shown in Fig. 5a. This appears to trigger the formation of amorphous bands that are seen in the experimental images (see, for example, Fig. 3d). Then the deconstructed icosahedra in the TB plane make it easier to form an amorphous band in the perfect B_6_O, as shown in Fig. 5b. The orientation of the amorphous band extends along the 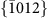 plane, which is not aligned along the TB plane but is consistent with the simulations of biaxial deformation of B_6_O described above.

The transition of the amorphous band from TB plane to 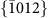 plane may relate to the complex stress conditions in nanoindentation experiments. Based on the above QM study, the amorphous band formed along the TB plane is under the biaxial stress conditions where the shear stress is along the TB plane and the compressive stress is perpendicular to the TB plane (Fig. 5a). As the amorphous band changes and propagates along the 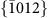 plane in the perfect B_6_O, the shear stress is still along the TB plane, whereas the compressive stress is along the 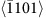 direction, as shown in Fig. 5b.

Our early study^17^ showed that nucleation of amorphous bands is possible in the perfect crystal, but our current experimental observation suggests that they are easier to nucleation in the vicinity of twins. This finding is in good agreement with the QM calculations that find the twinned structure to have a lower maximum shear strength by 7.5% compared with perfect crystal structure.

Figure 3b shows that the amorphous bands propagate across the twin bands without obvious turning. Our QM simulations on the biaxial shear deformation of the twinned structure find deconstruction of the icosahedra only in the TBs and the hard shear region, which is consistent with the TEM images shown in Supplementary Fig. 7 (white dots correspond to the central positions of the B_12_ icosahedral clusters). This suggests some discontinuity might exist in the twinned structure. However, the twin bands are too narrow to observe the discontinuity in our experimental images. Our current paper focuses on the nanoscale twins in B_6_O and their effects to the initiation of the amorphous band formation. It will be interesting to later examine the amorphous band propagation after initiation.

The QM calculations find that the amorphous band in B_6_O is along 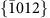 that is different from B_4_C, which is along 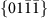
18. To compare the mechanism for these two systems, we performed biaxial shear simulations on B_4_C perfect crystal leading to the structural changes shown in Supplementary Fig. 8. For B_4_C, the deconstruction of icosahedra is still along 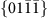 family plane because the failure of B_4_C arises from the interaction of the C–B–C chain with the B_11_C icosahedral cluster. In contrast for B_6_O, the C–B–C chain is replaced by two individual O atoms, which significantly changes the deformation and failure mechanism.

In this work, we identified the atomistic structure of nanoscale twins in B_6_O using a combination of TEM experiments and QM simulations. Then, we used QM to determine the shear deformation mechanism of twinned structures, finding that the twin can transform back to the crystalline structure. This is similar to the structural recovery in perfect B_6_O. These results indicate that pure shear deformation need not form an amorphous shear band for either perfect or twinned B_6_O.

Then, we investigated the deformation mechanism for twinned and perfect B_6_O under indentation conditions with QM biaxial shear simulations. Here we found that for the perfect crystal the layer deconstructs in alternate layers of 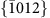 family planes, with a maximum shear stress of 39.9 GPa. In contrast, for the twinned system, the amorphous band forms more easily when shear is along the twin plane, with a maximum shear stress of 36.9 GPa. Thus, it is plausible that amorphous shear bands nucleate at the nanoscale twins, which is supported by indentation experimental observation of the co-existence of nanoscale twins and amorphous shear bands.

Summarizing, we demonstrate that combining STEM nanomechanical testing with QM modelling can provide a deeper understanding of the fundamental deformation mechanisms in ceramics, which may enable the design of ductile superhard ceramics.

## Methods

### Computational details

All simulations were performed used the Vienna Ab-initio Simulation Package (VASP) periodic code with plane wave basis sets^31^,^32^,^33^. We used an energy cutoff of 600 eV in all the simulations as it gives excellent convergence on energy, force, stress and geometries. We used the projector-augmented wave pseudopotentials for the Perdew–Burke–Ernzerhof exchange-correlation functional^34^. The energy error for terminating electronic self-consistent field and the force criterion for the geometry optimization were set equal to 10^−6^ eV and 10^−3^ eV Å^−1^, respectively. The Monkhorst–Pack grid (8 × 8 × 8) in the *k*-space was used in the unit cell geometry optimization. Later, a smaller grid (2 × 2 × 2) and larger supercell (2 × 2 × 2) were used in the biaxial shear deformations. The Monkhorst–Pack grid (2 × 4 × 1) was used for the twinned B_6_O simulations.

To examine shear deformation, we imposed the strain for a particular shear plane while allowing full structure relaxation for the other five strain components^35^.

We mimic the indentation process by applying biaxial shear deformation. Here we considered the normal compressive pressure beneath the indenter and constrained the ratios of normal stress (**σ**_**zz**_) to shear stress (**σ**_**xz**_) as **σ**_**zz**_=**σ**_**zx**_tan*Φ*, where *Φ*=68° for a Vickers indentor^36^. The residual stresses after relaxing the other strain components in both shear and biaxial shear deformations were less than 0.5 GPa.

### Material fabrication and nanoindentation

The fully dense B_6_O was synthesized using hot-pressing techniques at 2,173 K, 60 MPa for 1 h. The synthesis procedure is described in detail elsewhere^37^. We performed a series of indentations on the smoothly polished B_6_O surface at a constant loading force (2.0 N) and loading rate (14.0 mN s^−1^) using a dynamic ultra-microhardness tester (Shimadzu W201S) equipped with a Berkovich diamond indenter. The cross-sectional TEM specimens of the indented B_6_O were prepared by a lift-out technique^38^ using a FIB system (JEOL, JIB-4600F). Before the TEM observations, the TEM sample was further gently milled below 500 eV by a Fashone 1,040 Nanomill system to remove the damage layer on the sample surface without altering the specimen chemistry and structure.

### Transmission electron microscopy

We characterized the microstructure of as-synthesized B_6_O using a JEM-2100 F transmission electron microscope (JEOL Ltd.) equipped with double spherical aberration correctors (CEOS GmbH) for the image- and probe-forming lens systems. The annular bright-field STEM (ABF-STEM) images acquired a detector angle of ∼12–23 mrad and point-to-point resolution 0.1 nm. ABF-STEM image simulations were performed using a software package of Win HREM (HREM Research Inc).

## Additional information

**How to cite this article:** An, Q. *et al*. Nucleation of amorphous shear bands at nanotwins in boron suboxide. *Nat. Commun.* 7:11001 doi: 10.1038/ncomms11001 (2016).

## Supplementary Material

Supplementary Information Supplementary Figures 1-9.
