# Supplementary material for: Nucleation of amorphous shear bands at nanotwins in boron suboxide
Source: Nat Commun. 2016 Mar 22;7:11001. doi: 10.1038/ncomms11001 (PMC4804168; doi:10.1038/ncomms11001)
Supplement: Supplementary Information — Supplementary Figures 1-9. [file ncomms11001-s1.pdf]

Supplementary Figure 1

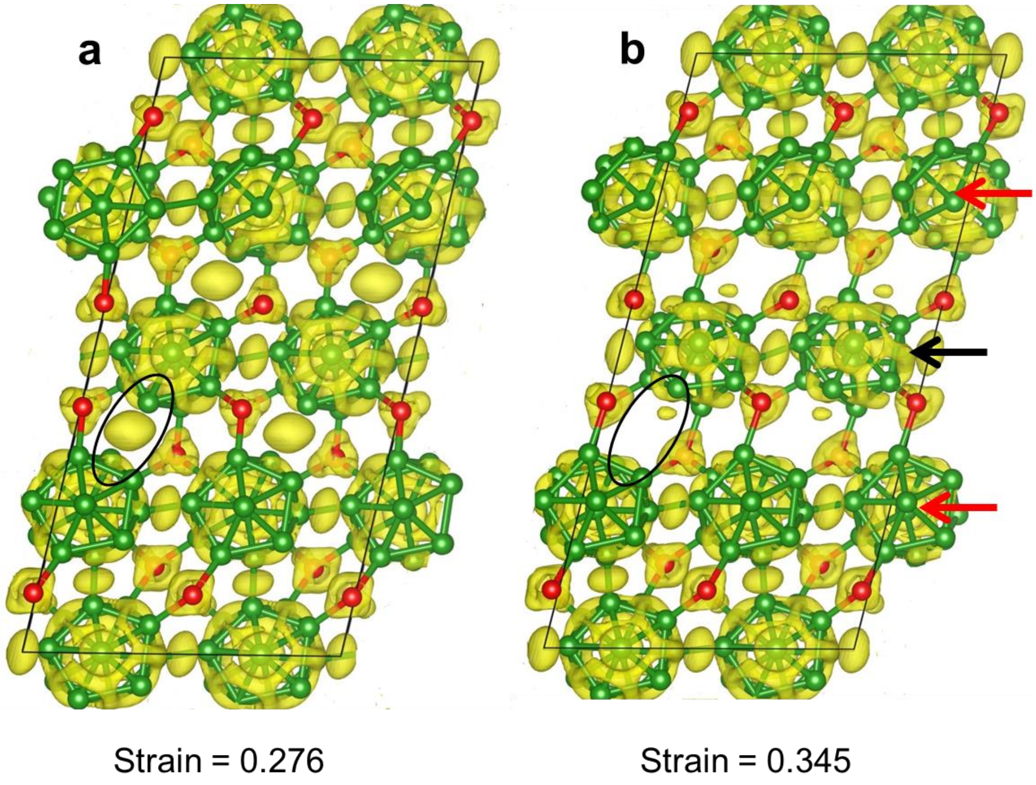

Supplementary Figure 1. **Structural changes and ELF of twinned B_6O for shear along the $\{0\bar{1}11\}$ twin plane.** (**a)** Structure at 0.276 strain, corresponding to the maximum stress status. The B-B bond in the oval is not breaking, indicated by the localized electrons at the bond. (**b)** Structure at 0.345 strain, before structure transformation. The icosahedra layer that break two B-B icosahedral bonds is represented by black arrow and the icosahedra layers that break one B-B icosahedral bonds are represented by red arrow.

Supplementary Figure 2

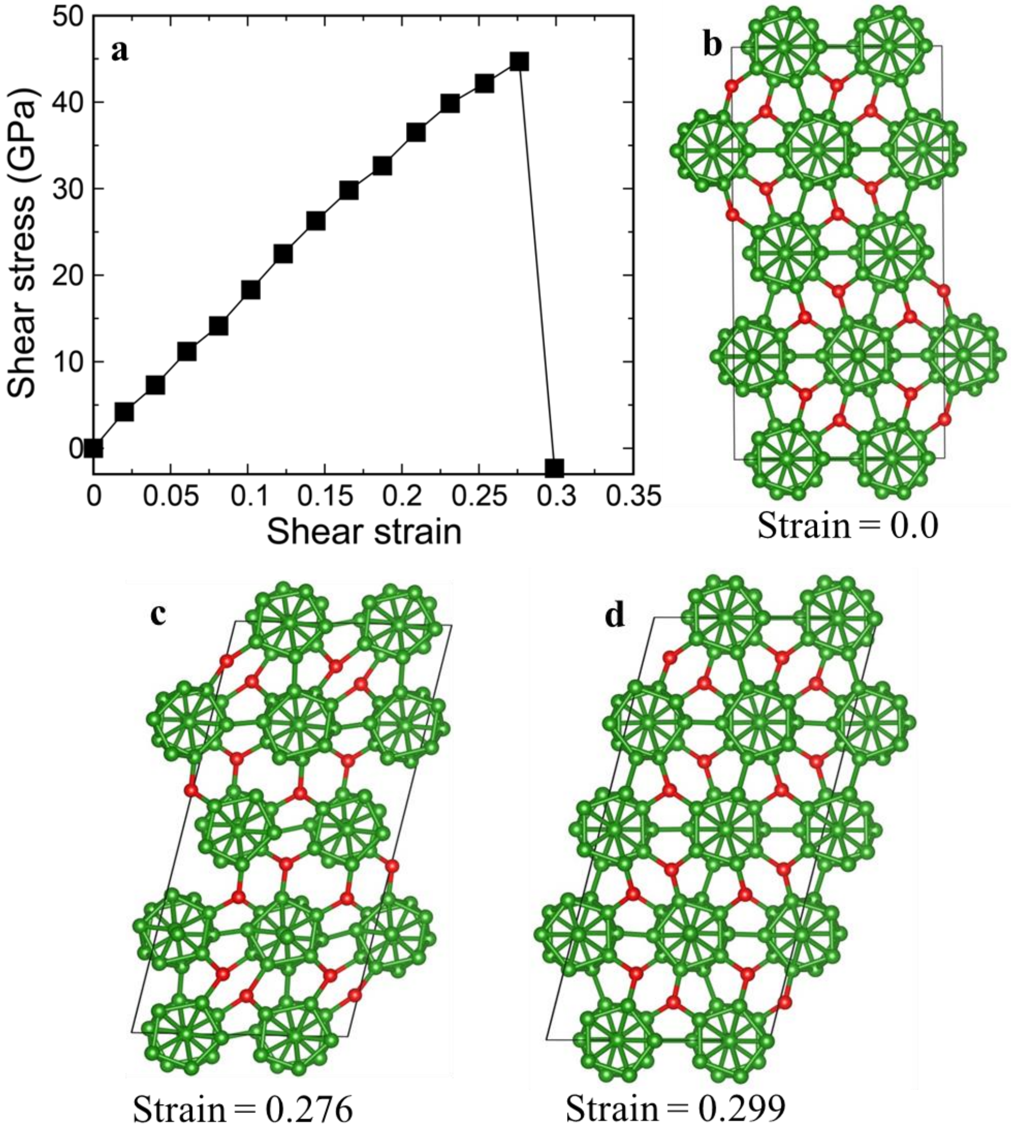

Supplementary Figure 2. The constant volume shear deformation of twinned B6O along $\{0\bar{1}11\}$ twin plane with the strain rate of 1.0 × 10^{10} s^{-1} using AIMD simulations. (**a**) Stress-strain relationship. (**b**) Initial twin structure at 0 strain. (**c**) Deformed twin structure before recovery at 0.276 strain. (**d**) Recovered twin structure at 0.299 strain.

Supplementary Figure 3

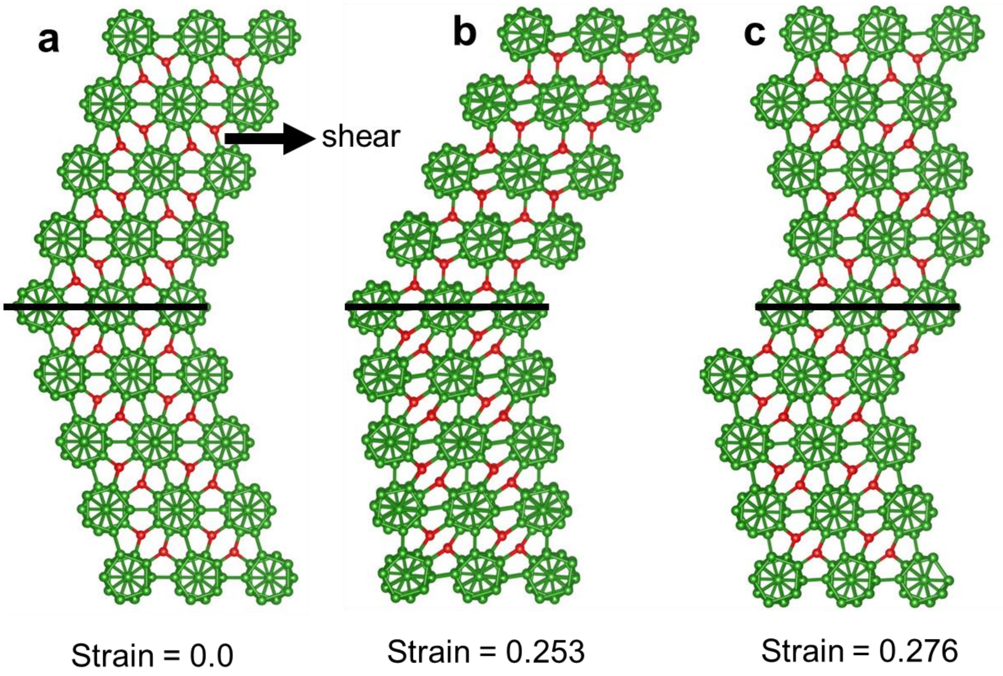

Supplementary Figure 3. **Structural evolution for the 4-layer twinned B_6O shearing along the $(0\bar{1}11)/\langle 10\bar{1}1\rangle$ slip system.** (**a**) 4-layre twinned structure. (**b**) Structure at 0.253 strain before failure. (**c**) Recovered structure at 0.276 strain. The TBs are indicated by the solid black line.

Supplementary Figure 4

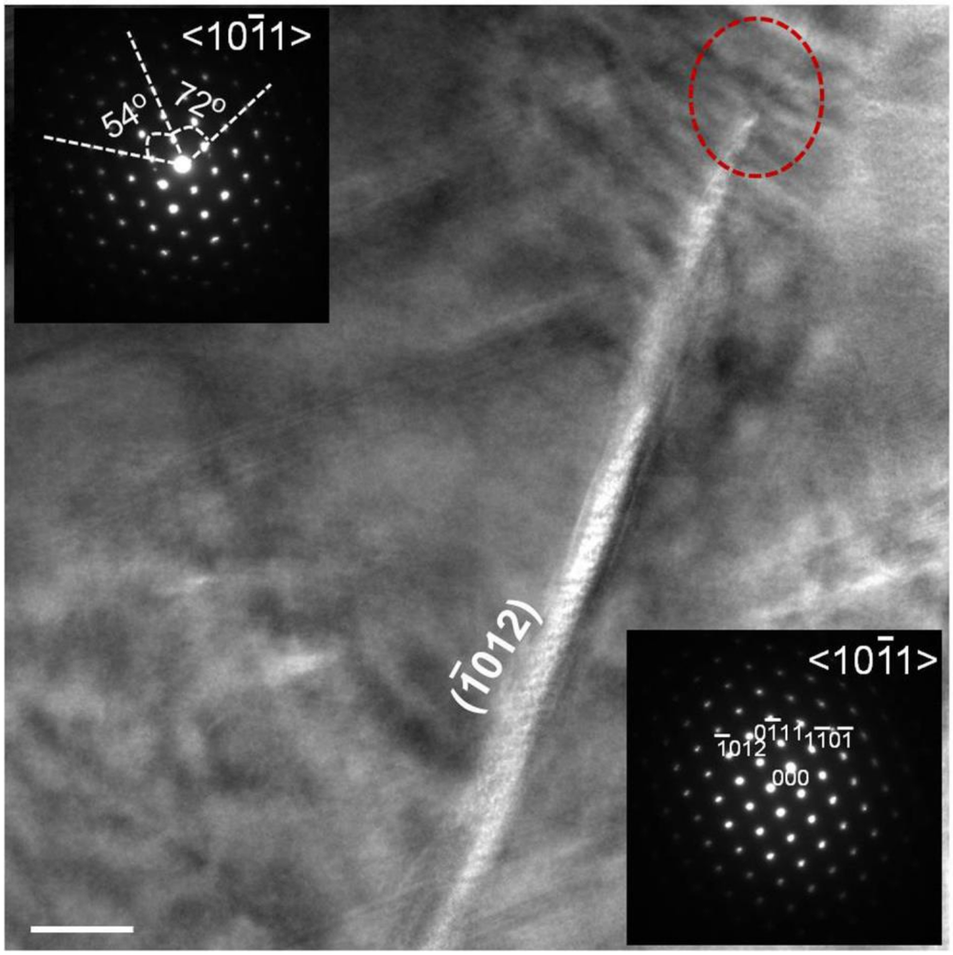

Supplementary Figure 4. Experimental observed amorphous shear bands within the deformed region. TEM image of the amorphous structure of the shear band with a width of 1-3 nm along the $\langle 10\bar{1}1 \rangle$ crystallographic projection. SAED patterns shown from crystalline region on either side of amorphous band. The angular mismatch in SAED patterns are less evidence by acquiring from the large area. The red dotted circle shows the initiation of amorphous band at the vicinity of twins. Scale bar is 20 nm.

Supplementary Figure 5

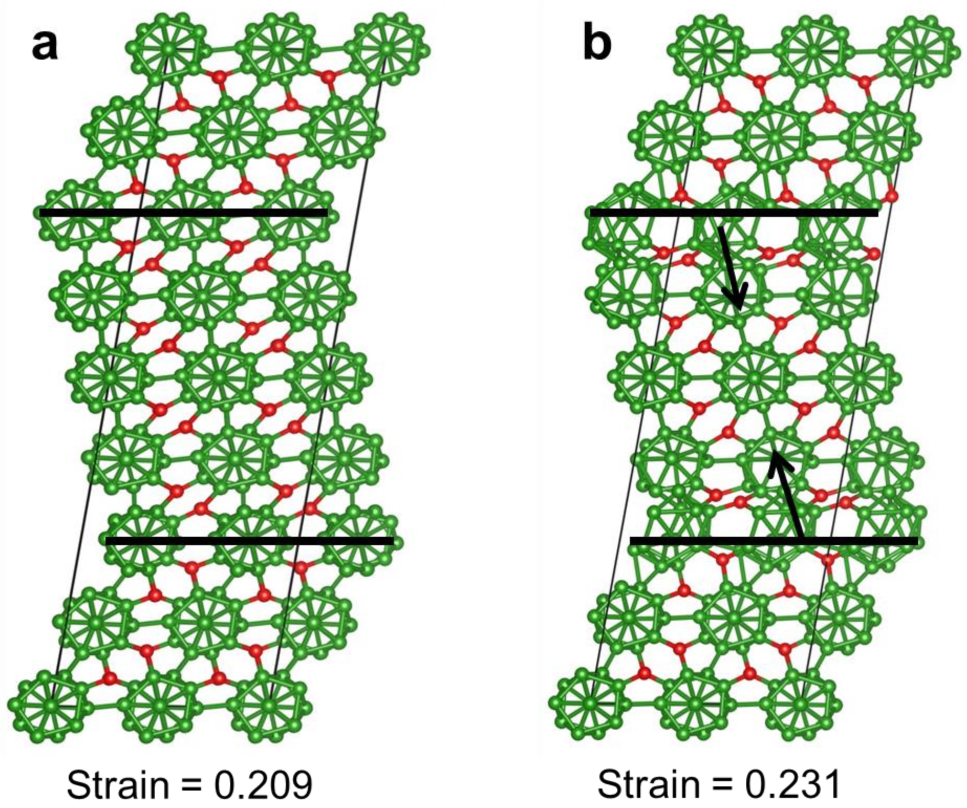

Supplementary Figure 5. **Structural evolution for 4-layer twinned B_6O under biaxial shear deformation along the $(0\bar{1}11)/<10\bar{1}1>$ slip system.** (**a**) Structure at 0.209 strain before failure. (**b**) Failure structure at 0.231 strain. The TBs are indicated by the solid black line. The arrows represent the deconstructed icosahedra that may lead to amorphous band formation beside the TB direction.

Supplementary Figure 6

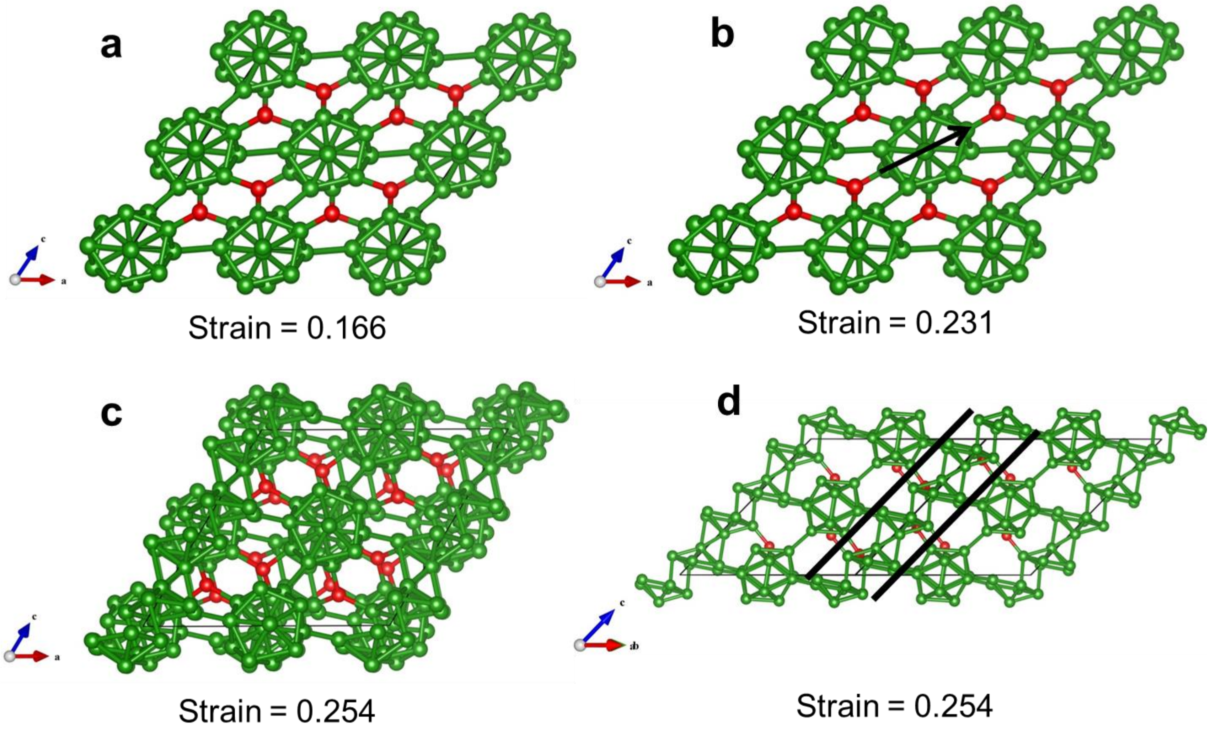

Supplementary Figure 6. **Structural evolution leads to amorphous band formation for perfect B_6O biaxial shearing along the $(0\bar{1}11)/<10\bar{1}1>$ slip system.** (a) Structure at 0.166 strain corresponds to the maximum shear stress. (b) Structure at 0.231 strain, before failure; the long axis of distorted icosahedra is represented by the black arrow. (c) Failure structure at 0.254 strain, viewed along the $[\bar{1}101]$ direction. (d) Failure structure at 0.254 strain, viewed along the $[2\bar{1}\bar{1}0]$ direction. The amorphous band is between the solid black lines.

Supplementary Figure 7

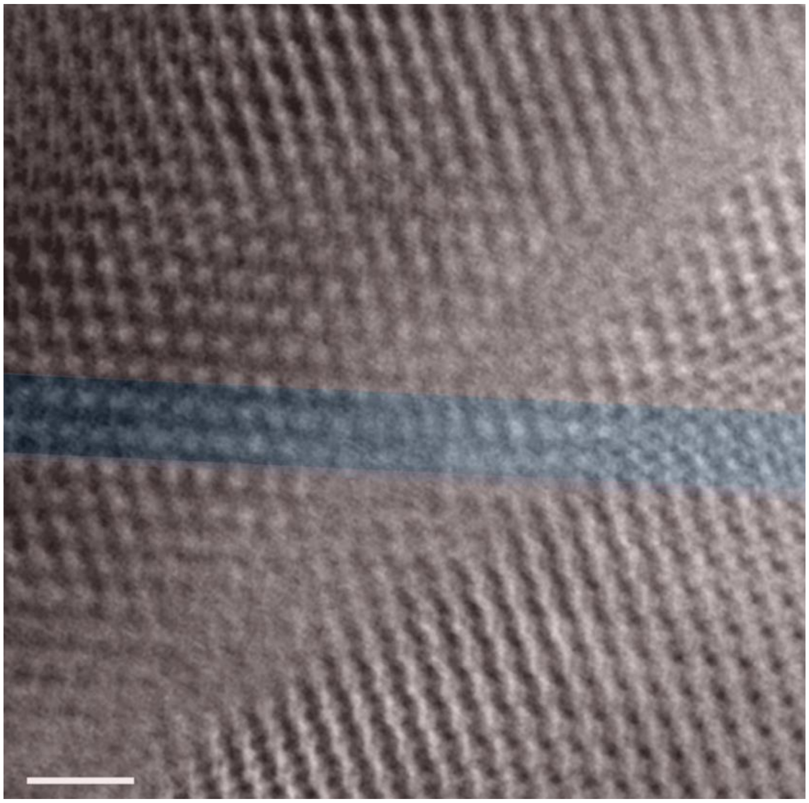

Supplementary Figure 7. STEM image showing amorphous shear band across the twin band. **S**TEM image shows the amorphous band cutting through the two-layer twin band. The icosahedra in the upper half twin layer are not deconstructed, which is consistent with the QM indentation experiments. scale bar is 1 nm.

Supplementary Figure 8

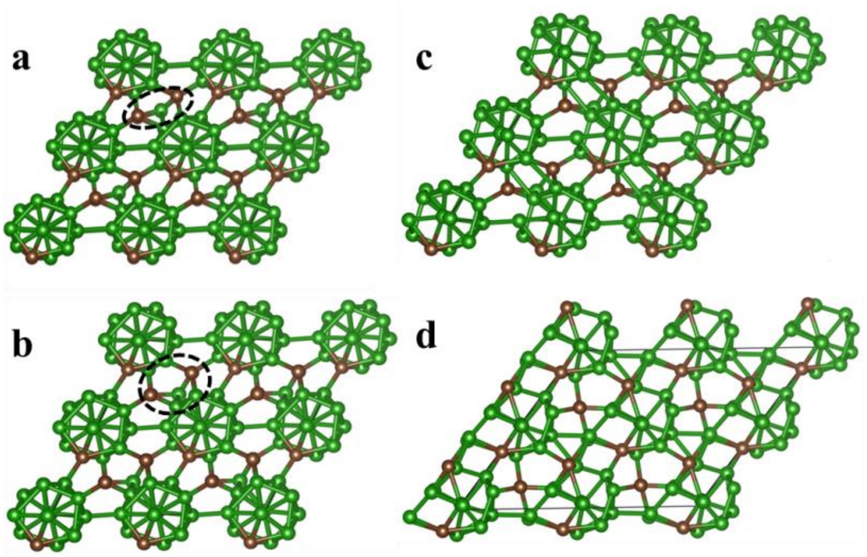

Supplementary Figure 8. The structural changes for perfect B_4C structures under biaxial shear deformation aimed at mimicking deformation under the indenter by imposing the relations $\sigma_{zz}=\sigma_{zx}\tan\Phi$. (**a**) Perfect B_4C at 0.136 strain before plastic deformation. (**b**) Perfect B_4C at 0.155 strain, where plastic deformation starts. (**c**) Perfect B_4C at 0.263 strain, before failure. (**d**) Perfect B_4C at 0.297 strain after failure.

Supplementary Figure 9

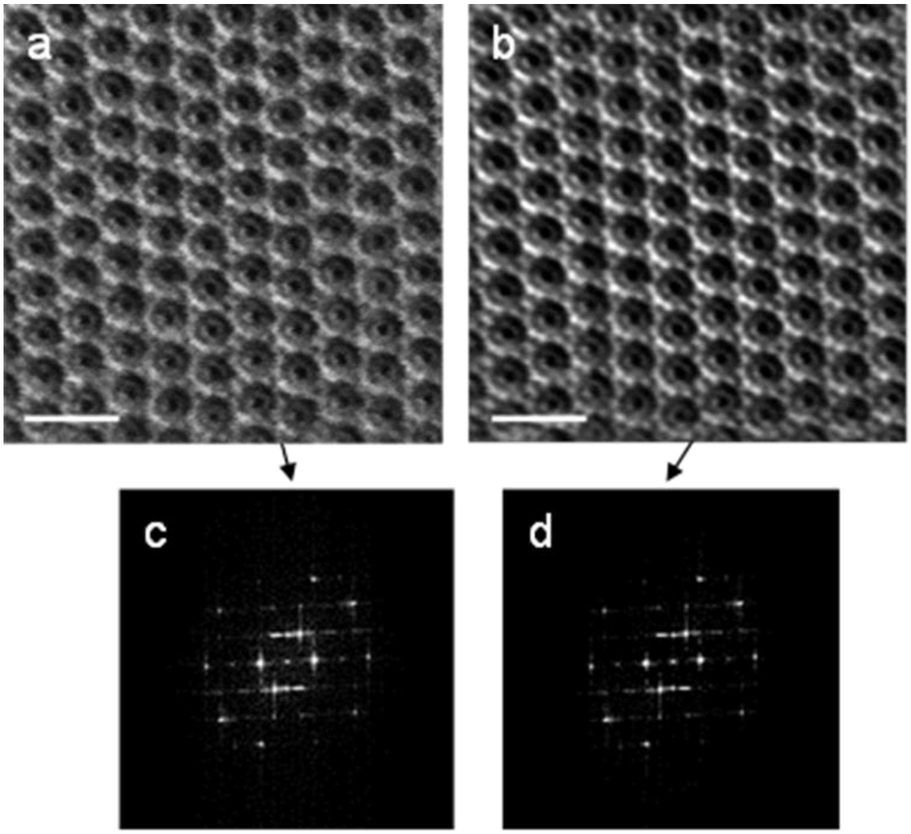

Supplementary Figure 9. **Examples of Fourier filtering to enhance visibility of B_6O atomic structure.** Annular bright field STEM (ABF-STEM) of B_6O (a) Raw and (b) Average Background Subtraction Fourier (ABSF) filtered images. (a,b) Scale bar is 1 nm. The background noise of ABF-STEM processed image is reduced as compared to the unprocessed data. (c and d) windows of Fast Fourier Transform (FFT) of raw and filtered image reflections.
